# A General Metric for the Similarity of Both Stochastic and Deterministic System Dynamics

**DOI:** 10.3390/e23091191

**Published:** 2021-09-09

**Authors:** Colin Shea-Blymyer, Subhradeep Roy, Benjamin Jantzen

**Affiliations:** 1 School of Electrical Engineering and Computer Science, Oregon State University, Corvallis, OR 97331, USA; sheablyc@oregonstate.edu; 2 Department of Mechanical Engineering, Embry-Riddle Aeronautical University, Daytona Beach, FL 32114, USA; ROYS5@erau.edu; 3 Department of Philosophy, Virginia Tech, Blacksbug, VA 24060, USA

**Keywords:** nonlinearity, model selection, chaos detection, change detection, model behavior mapping, dynamical similarity, causal discovery

## Abstract

Many problems in the study of dynamical systems—including identification of effective order, detection of nonlinearity or chaos, and change detection—can be reframed in terms of assessing the similarity between dynamical systems or between a given dynamical system and a reference. We introduce a general metric of dynamical similarity that is well posed for both stochastic and deterministic systems and is informative of the aforementioned dynamical features even when only partial information about the system is available. We describe methods for estimating this metric in a range of scenarios that differ in respect to contol over the systems under study, the deterministic or stochastic nature of the underlying dynamics, and whether or not a fully informative set of variables is available. Through numerical simulation, we demonstrate the sensitivity of the proposed metric to a range of dynamical properties, its utility in mapping the dynamical properties of parameter space for a given model, and its power for detecting structural changes through time series data.

## 1. Introduction

The term dynamical similarity refers to the degree to which the dynamics governing the evolution of a system over a period of time resembles the dynamics of another system, or the dynamics of the same system over a different period of time. Two fundamental problems of time series analysis are how best to measure dynamical similarity, and how to infer a given measure from noisy time series data. We address both problems, first by presenting a new metric of dynamical similarity that is indicative of underlying causal structure and that is well posed whether one is comparing two deterministic or two stochastic dynamical systems, and then by providing a range of tools for estimating this metric from data.

There is no uniquely best measure of dynamical similarity since the aptness of any given measure is relative to its intended use. However, there is broad interest in measures that are sensitive to the causal structure of a system in the sense of which variables directly determine the values (or stochastic distributions over the values) of other variables and with what functional form. For instance, the field of change detection is concerned with determining if and when the causal structure of a dynamical system has changed, as for example when an ecosystem (or climate) has been perturbed by external forcing, or when a mechanical component has begun to fail [1]. This amounts to determining whether one and the same system at a later time is dynamically similar to itself at an earlier time. Classically, this problem has been pursued under the assumption that the behavior of some stochastic system before and after a rapid change in parameter values is stationary [2], though progress has been made on the problem without the assumption of stationarity [3]. From the perspective of deterministic complex systems, the problem of change detection manifests as either the problem of detecting incipient bifurcations [4] or transitions between regions of the system’s attractor [5]. The more general problem encompassing both approaches is the detection of change in the structure of a dynamical system without assumptions of determinism or stationarity, such as detecting incipient bifurcation in an arbitrary stochastic dynamical system [6]. In general, we want to know whether a given system will continue to evolve and respond to perturbations in the same way as it did before.

It has been recognized for some time that it is often more important to know the degree of difference in dynamics after a change rather than the mere fact of change [7]. While change detection is often treated as a binary statistical decision problem [8], explicit measures of dynamical similarity have been used to assess whether a dynamical shift is practically (as opposed to statistically) significant [7]. Implicitly, such a notion of dynamical similarity plays a central role in system identification, where it is often important to know at the outset whether the system in question can be described with sufficient fidelity using a linear model [9], or in other words, whether the system is sufficiently dynamically similar to a linear one. Similarly, it is important to know the effective order of the dynamical process of interest in contexts where acquiring data points in a time series is expensive, such as community ecology, in order to know how long of a time series will be needed to fit a reliable model [10]. Relatedly, the degree of nonlinearity and chaos exhibited by a system is essential for managing error in system control or prediction [11,12,13,14]. Each of these problems—identifying effective order, nonlinearity, and chaos—can be seen in terms of dynamical similarity. Whether a system is strongly nonlinear is equivalent to asking whether and to what degree it is dynamically similar to a strongly nonlinear system and mutatis mutandis for effective order and chaos.

Whatever the appropriate measure of dynamical similarity, the problem of estimating its value from data is made more difficult with increasing sampling noise, and becomes significantly more challenging to address for stochastic systems. Model validation is notoriously difficult for stochastic systems [15,16]. Model validation amounts to assessing the similarity between one system—the model—whose dynamics is exactly characterized, and a target system with unknown dynamical properties; a valid model is one whose dynamics matches that of the target. The difficulty of this comparison is only compounded when one attempts to determine the similarity between two systems, both with unknown dynamics. Furthermore, existing techniques for detecting change or assessing, e.g., nonlinearity, are highly sensitive to sampling noise [2,17].

We here describe a general metric of dynamical similarity that is well posed for both stochastic and deterministic dynamical systems and which is sensitive to the effective order of dynamics, the degree of nonlinearity, and the presence of chaos. Importantly, this metric can be informative of these dynamical features even when only partial information about the dynamical state of a system is tracked, or a lossy function of the dynamical variables is observed, or in other words, if the system is only “partially observed” [18]. We introduce a variety of algorithms to show that this metric can be learned in a range of contexts, from situations in which one has full control of the dynamical system and complete dynamical information to situations in which only partial information is available for passively observed systems. We also demonstrate how this metric can be applied to the problem of change detection in this range of circumstances, and how it can be deployed to rapidly map out the varieties of dynamical behavior as a function of parameter values for a given dynamical model.

## 2. Related Work

### 2.1. Assessing Nonlinearity, Chaos, and Effective Order

The metric of dynamical similarity we describe is sensitive to the degree of nonlinearity in that the greater the magnitude of nonlinear terms in the governing differential equations of a system, the more it differs according to our metric from otherwise similar linear systems. Though not typically framed in terms of dynamical similarity, a number of measures of dynamical properties that could be used as such have been introduced previously for the purpose of detecting nonlinearity in time series data. In one prominent approach, introduced by Theiler et al. [19], one or another of these dynamical measures is deployed as a discriminative statistic for binary hypothesis testing where the null hypothesis is a linearized dataset—the surrogate data—constructed with a model that preserves properties such as the mean and variance of the original data. While Theiler et al. [19] used a battery of statistics familiar from dynamical systems theory, including the correlation dimension, Lyapunov exponent, and forecasting error, much recent work draws upon information theoretic constructs. Paluš [20,21], for instance, pursues the method of surrogate data using redundancy (the multidimensional generalization of mutual information). Unfortunately, redundancy and similar information theoretic measures reflect not just the dynamical relations among variables but also the apparent coordination imposed by their shared history, forcing function, or boundary conditions. Other information theoretic entropies have been pursued that better capture the causal relations among variables. The transfer entropy [22,23], for example, considers the probabilities of state transitions rather than of the states themselves. It has been applied as a discriminative statistic in the surrogate data framework by Nichols et al. [24], and was shown to outperform time-delayed mutual information. All of these methods, however, still depend upon the assumption that the system being assessed is at most weakly nonstationary. Merging information theoretic and dynamical systems approaches, Bandt and Pompe [25] introduced the permutation entropy, which they describe explicitly as a measure of the complexity of system dynamics. For a given *n* (typically chosen to be on the order of 10), the permutation entropy is an information theoretic entropy based on the probability of each of the n! permutations of the ordinal ranks of the elements in each *n*-sample long subsequence of a time series. It closely tracks the Lyapunov exponent λ, and requires that a time series exhibit only a very modest sort of stationarity. Typically, it is employed in the surrogate framework as a discriminative statistic [26], as is the related quantity known as the “number of missing ordinal patterns” [27]. More to the point, the permutation entropy allows for the sort of comparison between dynamical systems that our metric does, though with one substantial restriction: it vanishes uniformly for monotonic functions, whether nonlinear or not.

There is particular interest in distinguishing chaotic nonlinear systems from nonchaotic systems. Some methods for doing so require comparison with an explicit contrast class of models such as the method of comparing the predictive power of linear and nonlinear models of Volterra-Wiener form [28], and the method of surrogate data using the correlation dimension as the discriminative statistic [29]. Most, however, focus on some endogenous property of a system that can be estimated directly from a time series. Measures of this sort include (but are not exhausted by) Lyapunov exponents [30,31], the correlation dimension itself [32], nonlinear forecasting [33] (which provides an estimate of the largest Lyapunov exponent and has been found more robust than the correlation coefficient [34]), the determinism test [35,36], Kolmogorov entropy [37], the noise titration test [38], and the 0–1 test [39]. More recently, Kulp and Zunino [40] used the encoding scheme of Bandt and Pompe [25] to construct a “symbolic spectrum” in the manner of Yang and Zhao [41]. By examining the spectrum for a time series and looking for missing ordinal patterns with zero standard deviation (a hallmark of determinism), deterministic dynamics can be distinguished from stochastic, and by noting variation for some patterns, chaos can be distinguished from periodicity. Because hyper-chaotic systems have fewer forbidden patterns with zero variance, this test has the potential to yield an integral degree of chaos. With the exception of the 0-1 test, all of the other endogenous measures indicate a continuous-valued degree of chaos. Such a quantitative degree in turn admits an obvious measure of dynamical similarlity, at least with respect to chaos: the closer two systems are in their degree of chaos, the more dynamically similar they are.

The final feature of system dynamics with which we are concerned is the effective order or history dependence. Identifying the effective order of dynamics is an essential component of model selection in statistical forecasting and system identification. How such an identification can be made depends to a great deal on what is already known about the functional form of the connection between some finite number of past values of the system variables and the future values. Nonparametric system order identification techniques that can be applied to the more challenging classes of systems to which our algorithm can be applied, including nonlinear, stochastic systems, are diverse and well documented [42,43]. Broadly speaking, the problem is conceived as one of function estimation: the object is to find the form of a function f(·) and values of the associated parameters θ such that $y\left(t\right)=f(\vec{x}\left(t\right),\theta )+\epsilon \left(t\right)$, where y(t) is the target time series, $\vec{x}\left(t\right)$ is the input vector, and ϵ(t) is noise, typically presumed to be independent and identically distributed with constant variance [43]. As with function estimation in general, learning model structure—including model order—from a time series requires balancing the goodness of fit to the observed data and model complexity. There are a diversity of general approaches to achieving this balance, including cross-validation [44], stepwise selection (using measures such as AIC to bound complexity and regularize the search) [45,46,47], structural risk minimization [48], and LASSO [49]. A distinct approach is provided by methods for determining the embedding dimension in dynamical systems [50,51].

### 2.2. Dynamical Similarity and Change

One straightforward application of a measure of dynamical similarity is the problem of detecting a change in the behavior of a system over time. There are at least three specific versions of this general problem recognized in the literature. The first concerns detection of points or portions of a time series that are outliers with respect to an unknown but stationary (or slowly changing) distribution. This is a species of the generic anomaly or outlier detection problem [52,53]. The second problem is the detection of state change. From a statistical perspective, this means identifying points in a time series at which the parameters of a stationary or linearly changing distribution change suddenly [2,54]. From a dynamical perspective, this means detecting changes from one stable state to another as system parameters vary, or finding points at which an evolving system moves suddenly into a different region of its attractor [5].

The third problem of behavior change—and that which concerns us here—is the identification of a change in time of the dynamics of a system. Such a change may be due to a change in parameter values, a change in the functional form of relationships among variables, or even a change in the causal structure among the variables, and may occur fast or slow relative to the timescale of observation. Before, during, and after the change, the system need not exhibit a stationary distribution. In other words, the problem is to determine when a system at one time is dynamically different or distant from itself at an earlier time. A variety of approaches have been proposed for detecting changes of this sort. The most straightforward involve time series similarity measures. An overview and quantitative comparison of the similarity measures has been provided in [55] and more recently in [56]. These include distance measures such as the family of Minkowski distances (which include Manhattan and Euclidean distances) and the Mahalonobis distance [57] that treat each time series as a point in a high-dimensional space, as well as similarity indices constructed from correlation measures like Pearson’s cross-correlation coefficient [58]. While these approaches work directly with time series (though often normalized), related approaches involve an initial transformation of the series, e.g., by replacing the data matrix with the first few principal components (linear functions of the original variables) as a function of time [59], or shifting to the frequency domain by FFT [60,61]. The point of these transformations is generally some combination of dimension reduction and the increase in sensitivity to significant features. More recently, efficient algorithms have been introduced for computing the matrix profile, which replaces a time series with a series of values of the minimum Euclidean distance between a moving window and all similarly-sized sub-sequences [62]. In the resulting profile, anomalous segments of a time series are indicated by high profile values, while repeating motifs appear as minima. All of these metrics can be used as a measure of dynamical difference to detect changes in behavior. Unfortunately, none of these approaches to change detection can distinguish between dynamical changes and mere changes of state.

There are, however, alternative approaches in the literature that are specifically sensitive to dynamical structure. One family of methods attempts to detect a variety of symptoms of an incipient bifurcation, such as an increase in variance, slower recovery from perturbations and a consequent increase in autocorrelation [63]. Methods of detection often focus on trends in fit coefficients of autoregressive models [4,64]. A different sort of approach focuses on properties of the system attractor that are invariant for fixed parameter values, such as the recurrence plot or density function of visitation over cells in a discretized phase space or the fractal dimension [7,65,66]. Hively et al. [7], for example, define two measures on the reconstructed phase space of a system that provide a dynamical distance between a reference case time series and a test case. They first convert each univariate time series, $x_{i}$, into a sequence of integers, $s_{i}$ between 0 and S−1 via the function $\mathrm{INT}[S\left(x_{i}-x_{\min}\right)/\left(x_{\max}-x_{\min}\right)]$. If *d* is the embedding dimension of the reconstructed phase space, this effectively divides the phase space into $S^{d}$ hypercubes or “bins.” They then compute the empirical distribution functions $Q_{i}$ and $R_{i}$ reflecting the population of the *i*th bin for the reference time series and base time series, respectively. The measures of dynamical distance they introduce are: $\chi^{2}=\sum_{i}{\left(Q_{i}-R_{i}\right)}^{2}/\left(Q_{i}+R_{i}\right)$, and $L=\sum_{i}\left|Q_{i}-R_{i}\right|$. When applied to a simulated Bondarenko neuron model [67], both of these measures increase monotonically as a key parameter is varied through a region of known chaotic behavior. In other words, the degree of dynamical dissimilarity was shown to track known structural dynamical changes. These measures (and related “connected” variants) were also applied to EEG data as a tool for detecting incipient seizures.

### 2.3. Dynamical Kinds

Effective order, nonlinearity, and chaos are all aspects of the causal structure of a dynamical system. The existing tests contrived to assess these aspects consider a target system in isolation; the details of a particular system’s behavior are used to determine, e.g., the effective order of its dynamics, and only after the fact is it recognized that two systems share such causal features in common. The theory of dynamical kinds offers an alternative approach: by determining that two systems are of the same or different dynamical kinds, we learn whether they share any of these dynamical properties. Information about any one can then be transferred to the class.

Dynamical kinds were first defined in [68], and have since been applied explicitly to problems of model validation [69] and causal discovery [70]. The dynamical kinds theory partitions the space of dynamical systems into equivalence classes—dynamical kinds—on the basis of dynamical symmetry. A dynamical symmetry is an intervention [71,72]—an externally induced change in the values of some of the dynamical variables of a system—that commutes with the time evolution of the system ([69], p. 162):

**Definition** **1.** *Let t be the variable representing time, let V be a set of dynamical variables, and let *Ω* be the space of states that can be jointly realized by the variables in V. Let σ:Ω→Ω be an intervention on the variables in Int⊆V, and $\Lambda_{t_{0},t_{1}}$ the time-evolution operator that advances the state of the system from $t_{0}$ to $t_{1}$. The transformation σ is a dynamical symmetry with respect to time if and only if for all intervals Δt and initial states $\omega_{i}\in \Omega$, the final state of the system ${\tilde{\omega }}_{f}\in \Omega$ is the same whether σ is applied at some time $t_{0}$ and the system evolved until $t_{0}+\Delta t$, or the system first allowed to evolve from $t_{0}$ to $t_{0}+\Delta t$ and then σ is applied. This property is represented by the following commutative diagram:*

(1) ω˜i→Λt0,t0+Δω˜fσ↑σσ↑σωi→Λt0,t0+Δωf

Biological growth offers a simple illustration of the concept. For an exponentially growing population of bacteria, for which the population *x* changes according to dx/dt=rx, any transformation that scales the population by a positive constant *k* is a dynamical symmetry of the system—scaling by *k* and then allowing the bacteria to grow for Δt versus growing for Δt and then scaling the resulting population by *k* results in the same final population.

The composition of any two dynamical symmetries (by successive intervention on the variables of a system) is itself a dynamical symmetry, and for any given system, its dynamical symmetries typically exhibit nontrivial algebraic structure under composition. It is the collection of symmetries and their structure under composition that characterizes a dynamical kind [68].

Jantzen [73] provides a method for determining whether or not two physical systems with continuous deterministic dynamics belong to the same dynamical kind directly from time series data without first constructing dynamical models of either system. The method exploits two facts: (i) that a necessary condition for two systems to belong to the same dynamical kind is that they share all of their dynamical symmetries, and (ii) that for every state-determined system in the same dynamical kind, there is exactly one symmetry that maps the unique trajectory passing through one point in phase space, $\vec{x}$, to the trajectory passing through another point $\tilde{\vec{x}}$. That is, even if systems A and B exhibit different trajectories given initial conditions $\vec{x}$ or $\tilde{\vec{x}}$, the symmetry connecting these two trajectories for system A must be the same as that for system B if they belong to the same dynamical kind. Numerically estimating and then comparing these symmetries from time series data thus provides a sensitive test for the sameness of dynamical kind that is robust under significant sampling noise.

## 3. Theory and Algorithms

### 3.1. Stochastic Dynamical Kinds

Dynamical kinds partition the space of possible dynamical systems based on their causal structure. However, as defined above, sameness of dynamical kind is binary; it does not indicate the degree to which two systems that are not of the same kind differ in their causal structure. It also fails to apply to systems with stochastic dynamics. This latter problem can be addressed with a more expansive definition of dynamical kind. Previously, it has been suggested that the definition of dynamical similarity should be generalized to accommodate stochastic dynamics [69]. When restricted to dynamical symmetries with respect to time (other types of symmetry are considered in [69]), that proposed definition reduces to the following:

**Definition** **2.** *Let V be a set of random variables, *Ω* the set of states that can be jointly realized by the variables in V, and *Γ* the space of probability distributions over *Ω*. Let σ:Γ→Γ be an intervention on the variables in Int⊂V. The transformation σ is a dynamical symmetry with respect to time if and only if σ has the following property: for all initial joint distributions $\gamma_{i}\in \Gamma$, the final joint probability distribution over V, ${\tilde{\gamma }}_{f}\in \Gamma$, is the same whether σ is applied at time $t_{0}$ and then time evolution $\Lambda_{t_{0},t_{0}+\Delta }:\Gamma \to \Gamma$ evolves the joint distribution, or the system is first allowed to evolve over an interval *Δ*, and then σ is applied. This property is represented in the following commutative diagram:*

(2) γ˜i→Λt0,t0+Δγ˜fσ↑σσ↑σγi→Λt0,t0+Δγf

We accept this definition, and use it to develop a natural metric over dynamical systems that solves the problem of degree. Specifically, we present a metric based on Definition 2 that provides a well-grounded degree of causal difference, and show that it is sensitive to variations in linearity, effective order, and the presence of chaos.

### 3.2. Constructing a Metric of Dynamical Similarity

Consider a dynamical system described by *n* variables, some of which may be derivatives or time-lagged versions of other variables. The states of such a system can be represented by *n*-dimensional vectors $\vec{x}\in \Omega$. Let Γ be the space of probability density functions over Ω. We say that such a system is *stochastically state-determined* (SSD) if and only if the (possibly stochastic) dynamics of the system is such that the probability density $\gamma_{i}\in \Gamma$ over possible states at time $t_{i}>t_{1}$ is completely determined by the density $\gamma_{1}\in \Gamma$ over states at $t_{1}$. In other words, for an SSD system there exists a map, $\Lambda_{t_{1},t_{i}}:\Gamma \to \Gamma$ that, for any $t_{i}>t_{1}$ advances the probability density over states of the system from $\gamma_{1}\left(\vec{x}\right)$ to $\gamma_{i}\left(\vec{x}\right)$ (“random dynamical systems” in the sense defined in [74] are thus SSD, though we do not insist that SSD systems be measure preserving). According to Definition 2, any function, σ:Γ→Γ that commutes with this map is a dynamical symmetry of the system. More precisely, σ is a dynamical symmetry of a system if, for every $t_{i}>t_{1}$

(3) $$\sigma \circ \Lambda_{t_{1},t_{i}}\circ \gamma_{1}\left(\vec{x}\right)=\Lambda_{t_{1},t_{i}}\circ \sigma \circ \gamma_{1}\left(\vec{x}\right)$$

where $\sigma \circ \Lambda_{t_{1},t_{i}}\circ \gamma_{1}$ is the probability distribution that results from successive application of the maps $\Lambda_{t_{1},t_{i}}$ and then σ to the original distribution $\gamma_{1}$ (and similarly for $\Lambda_{t_{1},t_{i}}\circ \sigma \circ \gamma_{1}$). This property is represented in by the following commutative diagram:

(4) γ˜1(x→)=σ∘γ1(x→)→Λt1,tiγ˜i(x→)=σ∘Λt1,ti∘γ1(x→)=Λt1,ti∘σ∘γ1(x→)σ↑σσ↑σγ1(x→)→Λt1,tiγi(x→)=Λt1,ti∘γ(x→)

Consider two probability densities, $\gamma_{1}$ and $\sigma \circ \gamma_{1}$, connected by one such dynamical symmetry at time $t_{1}$. We call a time series $\vec{x}\left(t_{i}\right)$ *untransformed* if, for every $t_{i}$, it is the value of a random variable distributed according to $\gamma_{i}\left(\vec{x}\right)$. In other words, an untransformed time series is a time series for a system that evolves from an initial value selected according to the distribution $\gamma_{1}$. Similarly, we call a time series $\tilde{\vec{x}}\left(t_{i}\right)$*transformed* if, for every $t_{i}$, it is the value of a random variable distributed according to $\sigma \circ \gamma_{i}\left(\vec{x}\right)$. We focus on a particular probability density function that relates the time evolution of untransformed and transformed time series. Specifically, if one of *n* distinct times, $t_{i}$, in the evolution of the system is selected at random from a uniform distribution over the *n* possibilities, we seek the probability density that any untransformed time series exhibits a system state $\vec{x}\left(t_{i}\right)$ and any given transformed time series presents a system state $\tilde{\vec{x}}\left(t_{i}\right)$ at the same time. This joint density is given by:

(5) $$\begin{array}{cc} \gamma^{*}\left(\vec{x},\tilde{\vec{x}}\right) & =\sum_{i}\left(\mathrm{prob}.\;\mathrm{that}\;t=t_{i}\right)\left(\mathrm{prob}.\;\mathrm{of}\;\vec{x}\;\mathrm{given}\;\gamma_{i}=\Lambda_{t_{1},t_{i}}\circ \gamma_{1}\right)\left(\mathrm{prob}.\;\mathrm{of}\;\tilde{\vec{x}}\;\mathrm{given}\;{\tilde{\gamma }}_{i}=\sigma \circ \gamma_{i}\right)\\ \; & =\sum_{i}\frac{1}{n}\gamma_{i}\left(\vec{x}\right)\sigma \circ \gamma_{i}\left(\tilde{\vec{x}}\right). \end{array}$$

Denote the cumulative distribution corresponding to $\gamma^{*}$ by $cdf^{*}$. This distribution for any given dynamical system is shaped by its causal structure. In order to compare the degree to which dynamical structure differs between systems, our approach is to compare $cdf^{*}$. A suitable metric for doing so is the energy distance [75,76,77].

The energy distance for any two cumulative distributions, $cdf^{A}$ and $cdf^{B}$ over random variables $X^{A}$ and $X^{B}$ is defined as follows:

(6) $$D_{E}^{2}\left(cdf^{A},cdf^{B}\right)=2E\left[\left|\right|X^{A}-X^{B}\left|\right|\right]+E\left[\left|\right|X^{A}-X^{A^{\prime }}\left|\right|\right]-E\left[\left|\right|X^{B}-X^{B^{\prime }}\left|\right|\right],$$

where $X^{A^{\prime }}$ and $X^{B^{\prime }}$ are equivalent in distribution to $X^{A}$ and $X^{B}$. The square root of the right hand side of Equation (6), $D_{E}$, is a proper metric, and is zero if and only if $cdf^{A}=cdf^{B}$.

We define the *dynamical distance*, $D_{D}$, according to the identity

(7) $$D_{D}\equiv D_{E}\left(cdf^{*A},cdf^{*B}\right)={\left(2E\left[\left|\right|X^{A}-X^{B}\left|\right|\right]+E\left[\left|\right|X^{A}-X^{A^{\prime }}\left|\right|\right]-E\left[\left|\right|X^{B}-X^{B^{\prime }}\left|\right|\right]\right)}^{1/2},$$

where the random variable $X^{A}$ (equivalent in distribution to $X^{A^{\prime }}$) has values in the 2n-dimensional space of joint states $\langle {\vec{x}}^{A},{\tilde{\vec{x}}}^{A}\rangle$, and similarly for $X^{B}$. For two systems A and B, if at each time index *i*, $\gamma_{i}^{A}\left(\vec{x}\right)=\gamma_{i}^{B}\left(\vec{x}\right)$, then the difference between $\gamma^{A*}\left(\vec{x},\tilde{\vec{x}}\right)$ and $\gamma^{B*}\left(\vec{x},\tilde{\vec{x}}\right)$ and thus any difference between $cdf^{*A}$ and $cdf^{*B}$ must be due entirely to the action of the respective symmetries, $\sigma^{A}$ and $\sigma^{B}$. In that case, measuring the dynamical distance $D_{D}\left(cdf^{*A},cdf^{*B}\right)$ provides a quantitative comparison of the symmetries of systems A and B, and thus of the underlying casual structure that gives rise to them.

In general, an energy distance can be estimated by computing the sample mean for each of the expectation values on the right-hand side of Equation (6). For a sample of *p* points from system A and *q* from system B, there are $p^{2}$ pairwise distances for estimating $E\left[\left|\right|X^{A}-X^{A^{\prime }}\left|\right|\right]$, $q^{2}$ for estimating $E\left[\left|\right|X^{B}-X^{B^{\prime }}\left|\right|\right]$, and pq for estimating the cross-term, $E\left[\left|\right|X^{A}-X^{B}\left|\right|\right]$.

Accordingly, $D_{D}$ can be estimated using a sample of 2n-dimensional vectors,

$\langle {\vec{x}}^{A}\left(t_{i}\right),\tilde{\vec{}}x^{A}\left(t_{i}\right)\rangle$ from system A and $\langle {\vec{x}}^{B}\left(t_{i}\right),\tilde{\vec{}}x^{B}\left(t_{i}\right)\rangle$ from system B, where ${\vec{x}}^{A}\left(t_{i}\right)$ is the *i*-th point in a time series from system A beginning with an initial value ${\vec{x}}^{A}\left(t_{1}\right)$ drawn according to $\gamma_{1}^{A}\left(\vec{x}\left(t\right)\right)$ and ${\tilde{\vec{x}}}^{A}\left(t_{i}\right)$ is the contemporaneous *i*-th point in a time series from system A beginning with an initial value ${\tilde{\vec{x}}}^{A}\left(t_{1}\right)$ drawn according to ${{\tilde{\gamma }}_{1}}^{A}\left(\vec{x}\left(t\right)\right)$, and similarly for system B.

### 3.3. Temporal Scale Matching

The density $\gamma^{*}$ depends on both the time-evolution operators $\Lambda_{t_{1},t_{i}}$ and the particular dynamical symmetry, σ, that maps $\gamma_{i}$ to $\sigma \circ \gamma_{i}$. In general, two distinct systems will differ with respect to $\Lambda_{t_{1},t_{i}}$, and so despite beginning with the same distribution it is impossible to find later times $t,t^{\prime }$ such that $\gamma^{A}\left(\vec{x}\left(t\right)\right)=\gamma^{B}\left(\vec{x}\left(t^{\prime }\right)\right)$. However, if Λ is sufficiently smooth and the means of the two distributions overlap over some nonempty time interval, then it is possible to find *n* times $t_{i}$ and $t_{i}^{\prime }$ (such that $t_{i+1}-t_{i}=c_{1}$ and $t_{i+1}^{\prime }-t_{i}^{\prime }=c_{2}$ for some positive constants $c_{1},c_{2}$) for which $\gamma^{A}\left(\vec{x}\left(t_{i}\right)\right)\approx \gamma^{B}\left(\vec{x}\left(t_{i}^{\prime }\right)\right)$. In particular, one could sample each system over a sufficiently short interval of time relative to the natural scale of its dynamics in order to limit the evolution of each system to within acceptable deviation from the shared starting condition.

If the times $t_{i}=t_{i}^{\prime }$ are externally determined (as is often the case with data received for analysis by someone other than the experimenter or for data acquired by instrumentation with a fixed sampling period) but multiple time series are available from each system, then the same end can be achieved approximately by truncating or clipping one of the two sets of time series so as to effectively alter the time scale on which the corresponding system is sampled. We introduce an algorithm based again on an energy distance as defined in Equation (6). Suppose systems A and B are originally sampled at regular intervals at $t_{1},t_{2},\ldots ,t_{n}$, and for both systems, a number *s* of untransformed and a number *s* of transformed time series are provided. Then we seek the index *m* for either A or B such that the following condition holds. If each time series in the untransformed set (from A or B) is truncated at m<n and for each index *i* of the truncated set of time series and each index, $\mathrm{floor}(i\frac{n}{m+1})$, of the intact set of time series (from B or A) the energy distance between the sets of values at those indices is computed and the results averaged over *i*, there is no choice of *m* for either system A or system B that yields a lower average. The objective of clipping in this way is similar in spirit to the objective of dynamic time warping (to select a mapping of the indices of one time series to those of another that minimizes the sum of distances between the matched pairs) [78]. However, dynamic time warping requires that the first and last indices of the two time series compared coincide (leaving the overall time interval over which the system evolves intact), and unevenly alters the sampling interval throughout a given time series. Furthermore, dynamic time warping is not defined for ensembles of replicates from each system, while the energy distance is apt for comparing the similarity of $\gamma^{A}\left(\vec{x}\left(t_{i}\right)\right)$ and $\gamma^{B}\left(\vec{x}\left(t_{i}\right)\right)$ at each $t_{i}$ given multiple time series samples from *A* and *B*. We therefore use the average energy distance over time to find an appropriate cut point for one or the other ensemble of time series.

If the set of transformed time series replicates for each system are clipped to match their corresponding untransformed curves, then the difference in $D_{D}$ between the two systems is driven by the unknown dynamical symmetry, σ. For all numerical experiments reported here, we used the clipping algorithm expressed in Algorithm 1 when comparing time series from two systems. More specifically, we used Algorithm 1 to determine the optimal length at which to cut untransformed time series samples from *A* with the samples from *B* held fixed, and then repeated the process to determine the best length to cut samples from *B* with *A* held fixed. We ultimately clipped the data of whichever of the two systems resulted in the lowest value of $D_{D}$ after clipping.
**Algorithm 1:** Clipping for temporal scale matching.
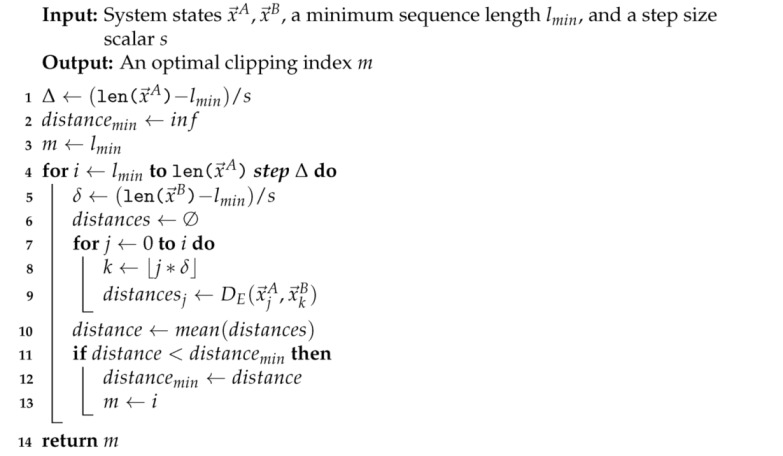


### 3.4. Partial Information and Degrees of Control

To make use of the dynamical similarity metric $D_{D}$ to compare physical systems, one needs an appropriate collection of time series. Ideally, one would obtain multiple untransformed and transformed time series for each system beginning with initial values identically and independently distributed according to $\gamma_{i}\left(\vec{x}\right)$ and $\tilde{\gamma_{i}}\left(\vec{x}\right)$, respectively, and where each system is sampled over a length of time such that for the pair of systems A and B to be compared, $\gamma_{i}^{A}\left(\vec{x}\left(t_{i}\right)\right)≊\gamma_{i}^{B}\left(\vec{x}\left(t_{i}\right)\right)$ at each time index *i*. If one has control over the initial conditions (the ability to intervene on the system), this is manageable. However, in many cases of interest, this is impossible. Furthermore, it is often the case that the variables in terms of which systems are described fail to satisfy the SSD condition because the set is incomplete or amounts to a noninvertible function of an SSD set. We describe methods for estimating our dynamical symmetry metric for every combination of these conditions.

#### 3.4.1. SSD Variable Set and Full Control of the Initial Distribution

At one extreme, we are provided full control over the initial conditions from which each time series for an SSD set of variables begins. In this case, the simplest approach is to take multiple time series samples from each system, half of which begin with an untransformed initial value of ${\vec{x}}_{0}$ and half of which begin at the transformed initial value, ${\tilde{\vec{x}}}_{0}\neq {\vec{x}}_{0}$. The clipping algorithm described above can then be applied and the dynamical distance $D_{D}$ estimated by using the resulting sets of time series to provide a sample of contemporaneous untransformed and transformed states, $\langle {\vec{x}}^{B}\left(t_{i}\right),{\tilde{\vec{x}}}^{B}\left(t_{i}\right)\rangle$, for each system.

#### 3.4.2. SSD Variable Set and No Control of the Initial Distribution

Even if a set of variables is known or suspected to be SSD, it is often or typically the case that time series are provided for analysis without the ability to manipulate the system to select specific initial values. In the most difficult case, only a single extended time series is available. While one cannot set the initial conditions of such a passively observed system, one can—under the additional assumption that the dynamics of the system are autonomous—imagine that subsequences within the given time series are each an initialization of that system. By carefully selecting such subsequences as instances of a system’s untransformed and transformed time series, it is still possible to estimate $D_{D}$.

**Figure 1:**
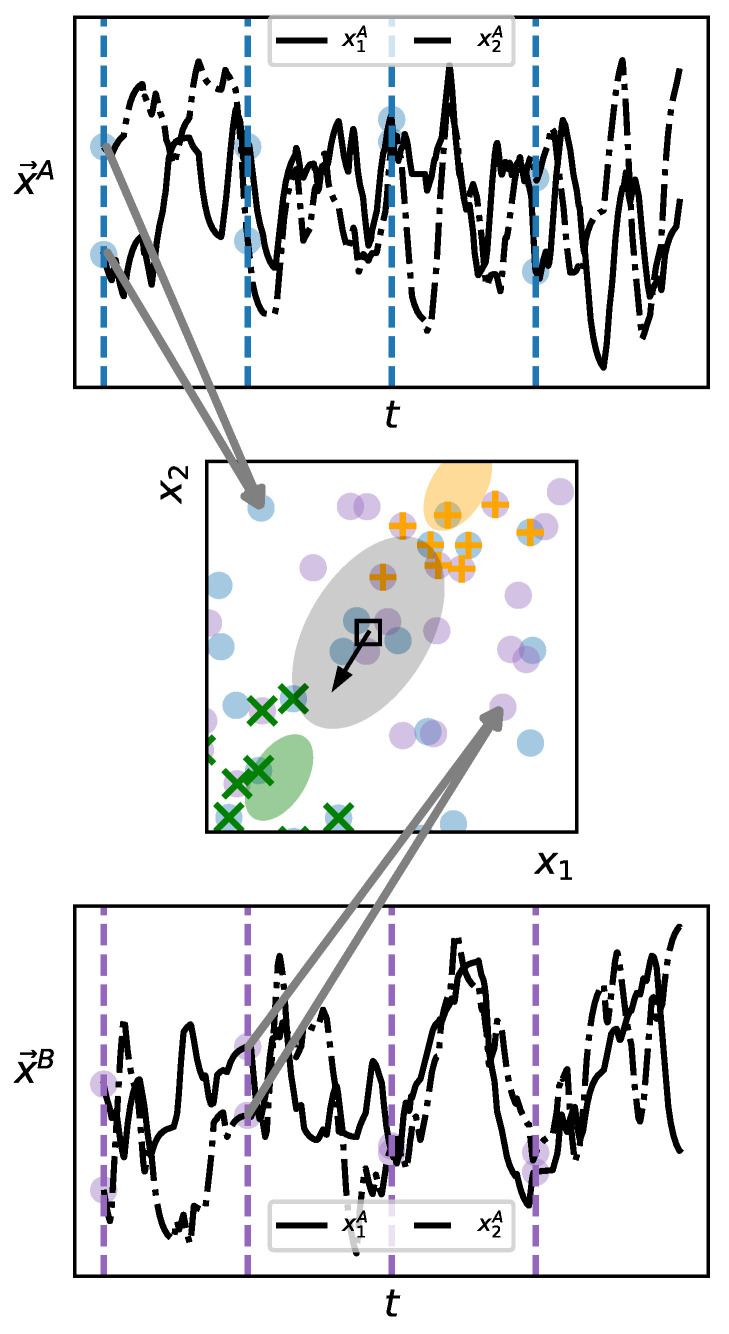
By fragmenting long time series, sets of appropriately selected fragments can serve as untransformed and transformed sets for estimating $D_{D}$. For two systems A and B, time series are divided into fragments of equal length (**top** and **bottom** plots), and the initial values of each segment from A and B are pooled in a common *n*-dimensional space (where *n* is the number of variables for each system) (gray arrows to **center** figure). For the pooled initial values, an overall mean is computed (black box, **center** figure) and the unit eigenvector $\vec{v}$ corresponding to the largest eigenvalue of the covariance matrix Σ is determined (black arrow, **center** figure). The major and semi-major axes of the gray ellipse at the center indicates the magnitude of the first and second singular values of Σ. Two new means are determined by moving in opposite directions along $\vec{v}$ (indicated by the centers of the green and orange ellipses), and two new covariance matrices constructed by scaling the original (indicated with the major and semi-major axes of the green and orange ellipses). Finally, a predetermined number of fragments are selected for inclusion in the untransformed and transformed sets (orange +’s and green ×’s respectively) for both systems by identifying initial values (points in the plane, center figure) with the highest density according to two *n*-dimensional normal distributions with the newly determined means and covariance matrices.

In describing the algorithm, we assume that two systems (A and B) are to be compared, but the method generalizes straightforwardly to arbitrarily many systems for which one seeks to estimate pairwise commensurable dynamical distances. First, the time series for both systems A and B are broken into subsequences of uniform length *l*. To select a subset of these sequences that will be treated as the untransformed set of replicates and the subset that will be treated as the transformed set for each system, we pool the initial values of all subsequences and compute the normalized eigenvector $\vec{v}$ with the largest eigenvalue λ for the covariance matrix Σ as well as the overall mean $\vec{\mu }$ of the pool. We then compute two new means, ${\vec{\mu }}_{\mathrm{untrans}}=\vec{\mu }-\alpha \lambda \vec{v}$ and ${\vec{\mu }}_{\mathrm{trans}}=\vec{\mu }+\alpha \lambda \vec{v}$ where α is a metaparameter controlling the degree of separation between the means of the two new distributions. Throughout, we have used a default value of α=1 unless otherwise noted. We compute the singular value decomposition of the original n×n covariance matrix $\Sigma =USV^{T}$ where *U* and $V^{T}$ are real, n×n orthogonal matrices and *S* is an n×n diagonal matrix with the singular values of Σ along the diagonal. We then construct a new covariance matrix, $\Sigma^{\prime }=U\left(\beta S\right)V^{T}$ where β is a second metaparameter determining the relative spread of the constructed distributions. We use β=0.2 unless otherwise indicated. Finally, for each system A and B, a set of untransformed replicates is chosen by selecting from the candidate subsequences those whose initial values have the highest density according to the *n*-dimensional normal distribution with mean ${\vec{\mu }}_{\mathrm{untrans}}$ and covariance matrix $\Sigma^{\prime }$. Likewise, a set of transformed replicates is selected based on their density according to the *n*-dimensional normal distribution with mean ${\vec{\mu }}_{\mathrm{untrans}}$ and covariance matrix $\Sigma^{\prime }$. This selection procedure is detailed in Algorithm 2. Once sets of replicates have been chosen in this way, the dynamical distance can be estimated as usual. This procedure is shown schematically in Figure 1 and presented in detail in Algorithm 2.
**Algorithm 2:** Choose untransformed and transformed representatives from data.
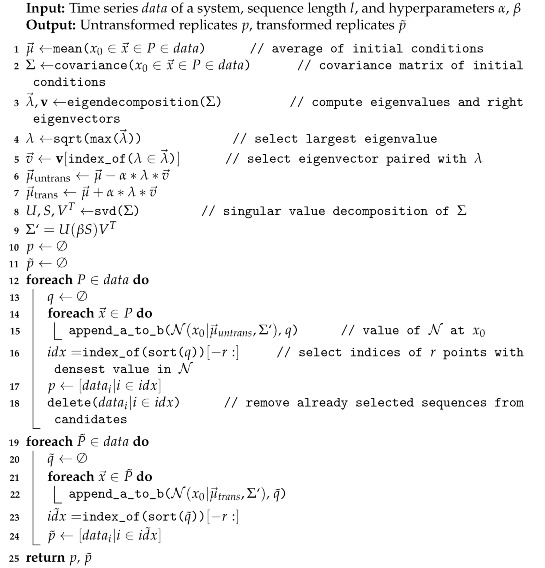


#### 3.4.3. Non-SSD Variable Set and Full Control of the Initial Distribution

Not every set of variables captures enough about the dynamically or causally relevant aspects of a physical system to determine future states (or distributions over future states) from the state (or distribution over states) at a given time. When dynamical variables go unobserved or when an observed set of variables is a lossy function of a complete set then the set of variables will generally fail to meet the SSD condition. We generically refer to such a set of variables as “partial” and the systems they describe as “partially observed.” While the methods described above for estimating $D_{D}$ do not work directly for partial variable sets, it is still possible in many circumstances to use the dynamical distance to discriminate among partially observed systems. For a system that is not SSD, the distribution over states $\gamma (\vec{x}\left(t\right))$ at a time *t* does not uniquely determine the distribution at a later time $t^{\prime }>t$. However, when the failure to meet the SSD condition is because the system is partially observed, there exists an unknown set of variables the states of which would uniquely determine $\gamma (\vec{x}\left(t\right))$ when supplemented by the observed variables. If transformed and untransformed time series can be obtained for each of two systems where the marginal distribution over the unobserved variables at $t_{1}$ is the same for both sets of untransformed series and the same for both sets of transformed series, then differences in the apparent values of $D_{D}$ will still be driven by the dynamical symmetries of the systems.

Given control of the initial state of each system to be compared, it is often the case that the procedure for setting the initial state in terms of the partial set of variables does fix a distribution over the unobserved variables. While this cannot be guaranteed, it can be tested given sufficient numbers of untransformed and transformed time series by verifying a fixed distribution over later times for a given initial (partial) state. The procedure for systems for which it is possible to intervene to set initial conditions but which are suspected of failing the SSD condition is thus to acquire multiple time series samples for each system for each initial condition, and then to estimate $D_{D}$ as above.

#### 3.4.4. Non-SSD Variable Set and No Control of the Initial Distribution

The most difficult scenario for accurately assessing dynamical similarity, regardless of the method used, is the case in which data are passively acquired such that there is no opportunity to set initial conditions and partially observed such that the given system of variables is therefore non-SSD. Even in this context, it is still possible to estimate $D_{D}$ at the price of some additional assumptions about the systems under consideration, namely that the all dynamical variables are bounded, that the dynamics is such that given sufficient time the system will pass nearby any system state previously observed, and that for any observed state there is a stationary distribution over states of the unobserved variables. Such a bounded system observed over a sufficiently long time will often exhibit an approximately stationary “distribution of distributions” that makes it appear SSD. For example, observing only the angular position of a pendulum does not determine its future position (angular position alone is not a state-determined set of variables). However, watching the pendulum swing for a while, one builds up a time series in which, from nonextremal positions, it moves left half the time and right half the time. So the system, though not state-determined, appears SSD. A similar situation obtains for stochastic dynamics.

Given that the available time series is long enough to meet these assumptions, then the dynamical distance $D_{D}$ can be estimated using the same algorithm as described in Section 3.4.2 for the case of a passively observed, SSD set of variables.

### 3.5. Change Detection

The methods for computing $D_{D}$ described above for conditions in which there is no control over the initial distribution can be used to detect changes in dynamics manifest in an extended time series. To identify such change points, a rolling window method can be used. For a window width of *w* and a lag (the space between the leading and trailing window) of *l* and a discrete time series, $\vec{x}\left(t_{i}\right),i\in \left\{1,\cdots ,n\right\}$, we treat the subsequences $\langle {\vec{x}}_{i},\ldots ,{\vec{x}}_{i+w}$ and $\langle {\vec{x}}_{i+w+l}\rangle ,\ldots ,{\vec{x}}_{i+2w+l}\rangle$ as time series from separate systems, and compute the value of $D_{D}\left(t_{i+w+l/2}\right)$ between them using the method of either Section 3.4.2 or Section 3.4.4, depending on whether the system is thought to be SSD. The appropriate window width depends upon how many replicates are required, and the minimum length of each. The best choice for the lag *l* will depend on the time over which the change in dynamical structure occurs. If *l* is shorter than the transition period, then both the leading and trailing windows will contain part of the transitional dynamics as they pass over the change, which tends to suppress the elevation of $D_{D}$, making it easier to miss the transition. If *l* is overly large, then it limits one’s ability to pinpoint the time of transition.

## 4. Numerical Experiments and Results

### 4.1. Difference in Dynamical Kind

We conducted numerical experiments with Lotka–Volterra ODE models and various derivatives designed to isolate one or another dynamical aspect in order to assess the extent to which $D_{D}$ is sensitive to linearity, effective order, chaos, and similarity of dynamical kind. For assessing sensitivity to dynamical kind as defined in Section 2.3, we numerically integrated a two-species instance of the general *n*-species Lotka–Volterra predator–prey system [79]:

(8) $$\frac{dx_{i}}{dt}=r_{i}x_{i}\left(1-\frac{\sum_{j=0}^{n}a_{ij}x_{j}}{k_{i}}\right),i=0,1,\ldots ,n,$$

where $x_{i}$ denotes the population size of the $i^{th}$ species, $k_{i}$ denotes its carrying capacity, $r_{i}$ denotes the intrinsic growth rate of the species, and $a_{ij}$ is the interaction coefficient of species *j* on species *i*. Any two systems related by scaling all $r_{i}$ by a positive constant share the same dynamical symmetries and thus belong to the same dynamical kind. Conversely, scaling the carrying capacities $k_{i}$ results in a different dynamical kind [73].

**Figure 2:**
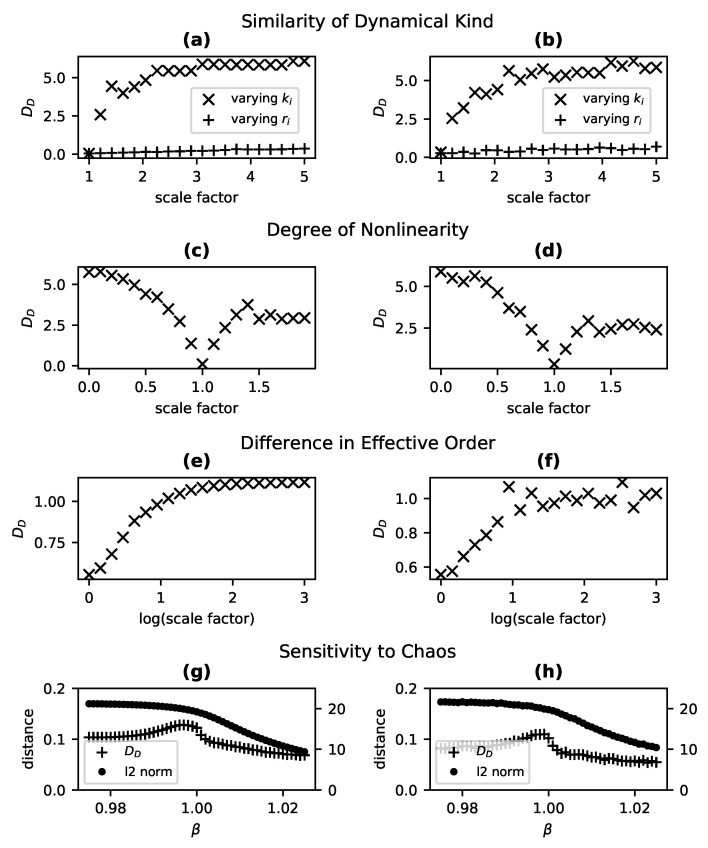
Sensitivity of $D_{D}$ to similarity of dynamical kind is demonstrated for two-species competitive Lotka–Volterra systems measured without sampling noise (**a**) or with normally distributed sampling noise with μ=0 and σ=5) (**b**). Relative to a system with growth rates of $\vec{r}=\left[1,2\right]$ and carrying capacities, $\vec{k}=\left[100,100\right]$, $D_{D}$ increases as the carrying capacities $\vec{k}$ of the comparison system are multiplied by an increasing scaling constant such that the systems belong to diverging dynamical kinds (×), but remains approximately 0 as the growth rates $\vec{r}$ are scaled (+), which is a dynamical symmetry connecting systems of the same dynamical kind. When sensitivity to nonlinearity is assessed by multiplying the interaction matrix by a scale factor relative to the reference system with α=[[1,0.5],[0.7,1]], giving a linear system at a scale factor of 0, $D_{D}$ increases as the nonlinearity of the comparison system is increased (**c**), even in the presence of sampling noise (**d**). $D_{D}$ also increases between systems as their effective order diverges. For a modified Lotka–Volterra system that is second order with a scale factor of 1 and approaches first order as that scale factor tends to infinity, $D_{D}$ increases rapidly from 0 relative to a reference system with a scale factor of 1, with sampling noise (**e**) and without (**f**). $D_{D}$ also responds specifically to chaos, rising relative to a nonchaotic reference system of similar nonlinearity and order as a four-species Lotka–Volterra system passes through a chaotic transition in parameter space along the β direction (defined in Equation (11)) (+), both with (**g**) and without (**h**) sampling noise. The $l^{2}$ norm (·), on the other hand, changes monotonically across the chaotic transitions.

In order to demonstrate that the metric $D_{D}$ provides a degree of difference in dynamical kind (and so generalizes the binary decision process of [73]), we use a reference system A with ${\vec{r}}^{A}\equiv \left[r_{1}^{A},r_{2}^{A}\right]=\left[1,2\right]$, ${\vec{k}}^{A}\equiv \left[k_{1}^{A},k_{2}^{A}\right]=\left[100,100\right]$ and a comparison system B with ${\vec{r}}^{B}=s_{r}*{\vec{r}}^{A}$ and ${\vec{k}}^{B}=s_{k}*{\vec{k}}^{A}$. For each test, we generate two time series for each system: an untransformed series with an initial population of $\vec{x}=\left[5,5\right]$, and a transformed series with an initial state of [8,8]. When $s_{r}$ is fixed at a value of 1 and $s_{k}$ is varied for system B, $D_{D}$ increases with increasing $s_{k}$ (Figure 2a) and thus indicates divergence in dynamical kind, as expected. The same relationship is apparent in Figure 2b, for which the experiment was repeated using time series to which normally distributed noise with a mean of 0 and standard deviation of 5 (equivalent to 5% of the dynamical range of the system) has been added. Note that $D_{D}$ approaches a maximum value. For any dynamical system for which states are bounded (such that there is some c>0 for which $|\vec{x}|<c$), $D_{D}$ is bounded from above.

### 4.2. Nonlinearity

Of broader interest is the sensitivity of the proposed metric $D_{D}$ to the degree of linearity of one system relative to a benchmark system. To assess this aspect of performance, we contrived a modification of the Lotka–Volterra equations (Equation (8)) that allows us to modulate the system’s degree of nonlinearity:

(9) $$\frac{dx_{i}}{dt}=r_{i}x_{i}\left(1-\frac{\sum_{j=0}^{n}a_{ij}lx_{j}}{k_{i}}\right),i=0\ldots n\;$$

where the factor *l* scales the degree of nonlinearity of the system. When *l* is 0, the system is perfectly linear, while the nonlinear term dominates for large values of the scale factor *l*. We numerically integrated two parameterizations of a two-species version of this model, system A and system B. Both simulated systems are provided with identical growth rates and carrying capacities ($\vec{r}=\left[1,2\right],\vec{k}=\left[100,100\right]$). System A’s value of *l* was fixed at 1, while the value of the scale factor *l* for system B was varied from 0 to 1.8. As before, the differential equations were numerically integrated from t=0 to 15. Each system’s untransformed initial population is 5 members, and each transformed initial population is 8. The calculation of $D_{D}$ was repeated for an experiment using identical systems but for which normally distributed observation noise (σ=5) was added.

As seen in Figure 2c,d, $D_{D}$ goes to 0 where the nonlinearity factor is 1 and both systems are identical. It reaches a maximum when the nonlinearity factor is 0, and thus one system is fully linear while the other involves substantial nonlinear interaction. As the value of *l* increases above 1, $D_{D}$ appears to increase asymptotically as the nonlinear term comes to dominate. The dynamical distance is thus sensitive to relative decreases and increases in effective linearity of dynamics for this sort of system.

### 4.3. Effective Order

The effective order of a system determines its dependence on prior history and the complexity of boundary conditions necessary to forecast the system. Isolating order required constructing a second order version of the Lotka–Volterra equations (Equation (8)) outfitted with a scale factor (ω):

(10) $$\frac{1}{\omega }\frac{d^{2}x_{i}}{dt^{2}}+\frac{dx}{dt}-r_{i}x_{i}\left(1-\frac{\sum_{j=0}^{n}a_{ij}x_{j}}{k_{i}}\right)=0$$

As ω approaches infinity, the system approaches first order dynamics. In this test, the parameters of systems A and B are the same as the systems in Section 4.2, but for the absence of *l* and the presence of ω. System A, whose ω value set to 1, was compared to system B as its ω value grew exponentially. These systems were also run from t=0 to 15 for our tests.

We expect to see that systems with diverging effective order are farther apart according to the metric $D_{D}$. This expected pattern is evident without sampling noise (Figure 2e) and with sampling noise (Figure 2f). The distance between the two systems grows with the order factor, leveling out as the large ω value causes system B to approximate first order dynamics.

### 4.4. Chaos

Testing the sensitivity of our distance metric to the presence of chaos requires us to compare a known chaotic system to similarly parameterized systems that, so far as possible, hold constant the effective order and degree of nonlinearity. Ideally, one would identify a single parameter that can be varied to move through the chaotic region of parameter space.

To meet these constraints, we use a four-species Lotka–Volterra system described by Equation (8) (n=4). This system is known to exhibit chaos for a range of parameterizations, and a portion of this parameter space was explored in [80]. The latter identify three chaotic points in parameter space, labeled $\left(R_{1},A_{1}\right),\left(R_{2},A_{2}\right)$, and $\left(R_{3},A_{3}\right)$, where each $R_{i}$ is a particular vector of intrinsic growth rates ($\vec{r}$ in our notation), and the $A_{i}$ are interaction matrices (*a* in our notation). A 2-D plane in parameter space is then defined by linear combination of these points determined by a pair of coefficients α and β as follows:

(11) $$\left(R,A\right)=\left(R_{1},A_{1}\right)+\alpha \left(R_{2}-R_{1},A_{2}-A_{1}\right)+\beta \left(R_{3}-R_{1},A_{3}-A_{1}\right)$$

In our experiments, we chose a nonchaotic reference system at α=0.2,β=1.0 and computed $D_{D}$ relative to systems along a slice through this parameter space from α=0,β=0.975 to α=0,β=1.025. For these experiments, carrying capacities are fixed at 1 for all species (equivalent to expressing each population as a fraction of its carrying capacity). The untransformed time series begin with all species at a population of 0.1, and 0.2 for the transformed time series. The result in the absence of noise (Figure 2g, + marks) is a clear rise in $D_{D}$ in the vicinity of β=1 (where the behavior is known to be chaotic) with relatively constant distance over variations in β on either side of the chaotic (and near-chaotic) region. When the experiments are repeated with normally distributed observation noise, N(0,0.05), added to each time series, a nearly identical pattern is observed, albeit shifted to a lower mean value of $D_{D}$ (Figure 2h, + marks).

As a benchmark, we also computed the $l^{2}$-norm between the reference and comparison time series (specifically, the untransformed time series) for each value of β (Figure 2g, • marks). This provides a metric sensitive to differences in the gross shapes of trajectories. The $l^{2}$ norm declined monotonically over the chaotic region, providing no indication that such a transition has taken place, regardless of whether or not sampling noise was included.

### 4.5. Model Mapping: Lotka–Volterra

A generic metric of dynamical similarity provides a common yardstick for comparing arbitrarily many systems in a model-free way. Consequently, it offers the possibility of mapping out a portion of the parameter space for a system of interest, summarizing the overall similarity in dynamical behavior between any two such points. To demonstrate the efficacy and utility of such a unique dynamical map, we computed values of $D_{D}$ for all pairs of parameter values in a discretization of the parameter space of the four-species Lotka–Volterra system described in Equation (8) and explored by [80].

Untransformed and transformed time series samples are collected from regularly arranged points in the rectangular region of parameter space spanned by values of α between 0 and 1.2, and β from −0.2 to 1.2, with 50 equally spaced divisions along each axis. For all systems we sample, we set the carrying capacity of each species to 100 and the initial populations for all species to 5 for the untransformed systems, and to 8 for the transformed systems. Each system is simulated for 4000 time steps, with a Δt of 0.5.

The result of these computations is a 2500×2500 distance matrix. To visualize the similarity relations implied by this matrix, we deploy multidimensional scaling (MDS) to embed each point of our discrete parameter space in a continuous three-dimensional geometric space. Positions in this space are then normalized (such that positions in all three directions are within the interval [0,1]), and interpreted as colors in a standard color space (RGB color space). The map is thus constructed by coloring each cell of the 50×50 grid in the α−β parameter plane according to the color assigned by the MDS embedding. The result is a map of parameter space for which the hue of any one pixel is meaningless, but for which similarity of color corresponds to dynamical similarity, i.e., systems that are dynamically indistinguishable according to $D_{D}$ are shown with the same color. In other words, the closer two pixels are in hue, the closer they are in the dynamical space defined by $D_{D}$.

**Figure 3:**
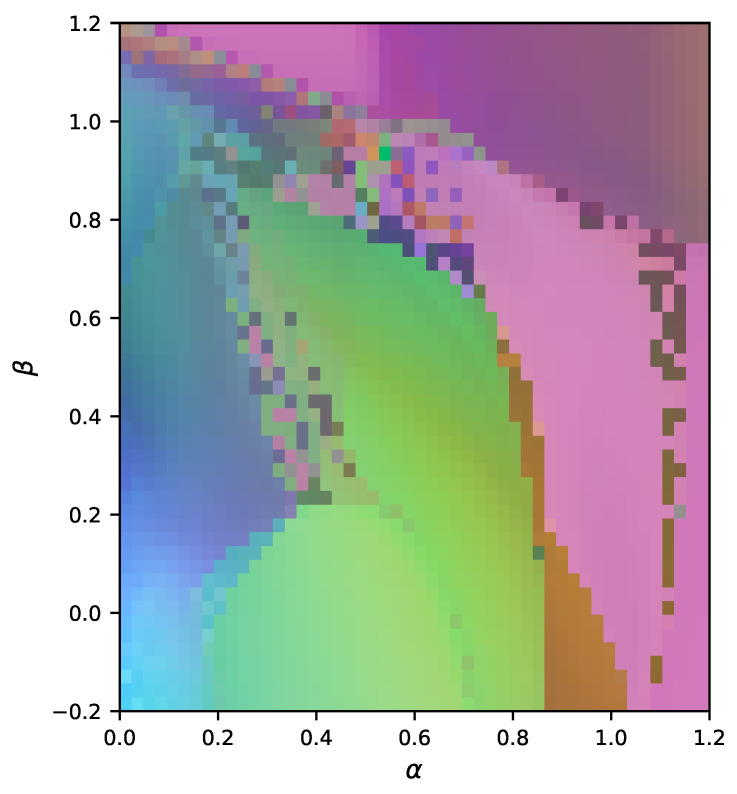
Exploration of the parameter space of a four-species Lotka–Volterra model in the same α-β plane explored in [80] and defined in Equation (11). The absolute hue of a pixel is meaningless. However, the closer two pixels are in color, the smaller the dynamical distance $D_{D}$ between them. This color mapping is achieved by using multidimensional scaling and the distance matrix of all pairwise $D_{D}$ values to embed every point of the depicted parameter space in a three-dimensional space that is then interpreted as RGB color space.

The resulting map (Figure 3) can be directly compared with that in [80]. The latter colored the plane in parameter space based on the number of species remaining at steady-state. The dynamical similarity map captures much of the same broad structure, but reflects significantly more nuance since the mere number of species at equilibrium is a poor indicator of, e.g., effective order or degree of nonlinearity. Note that the chaotic points at (0,0),(0,1) and (1,1) do not stand out as distinct from surrounding points. This is likely due to the coarse grain of our map and the extreme narrowness of the chaotic regions.

Using a low-dimensional embedding, constructed without normalization, we have discovered well-structured regions of similar dynamical structure, demonstrating the application of our algorithms as a novel method for exploring a system’s behavior.

### 4.6. Stochastic Dynamics and Partial Systems

The simulation experiments described above all involve state-determined systems, with or without measurement noise. However, $D_{D}$ applies to and can be estimated for systems that are only SSD. Furthermore, as described in Section 3.4.3, $D_{D}$ can be estimated for systems that are SSD but only partially observed. To assess the effectiveness of these estimation procedures, and the sensitivity of $D_{D}$ for SSD systems that are not state-determined, we constructed a stochastic version of the Lotka–Volterra equations based on [81]:

(12) $$\Delta x_{i}\left(t\right)=x_{i}\left(t\right)r_{i}\left(t\right)\left(1-\sum_{j=0}^{n}a_{ij}x_{j}\left(t\right)\right)\Delta t+\sigma_{i}x_{i}\left(t\right)\xi_{i}\left(t\right)\sqrt{\Delta t}+\frac{\sigma_{i}^{2}}{2}x_{i}\left(t\right)\left({\left(\xi_{i}\left(t\right)\right)}^{2}-1\right)\Delta t,$$

where $\sigma_{i}$ is the intensity of the noise for species *i*, and $\xi_{i}$ is a Gaussian random variable of the form N(0,1).

**Figure 4:**
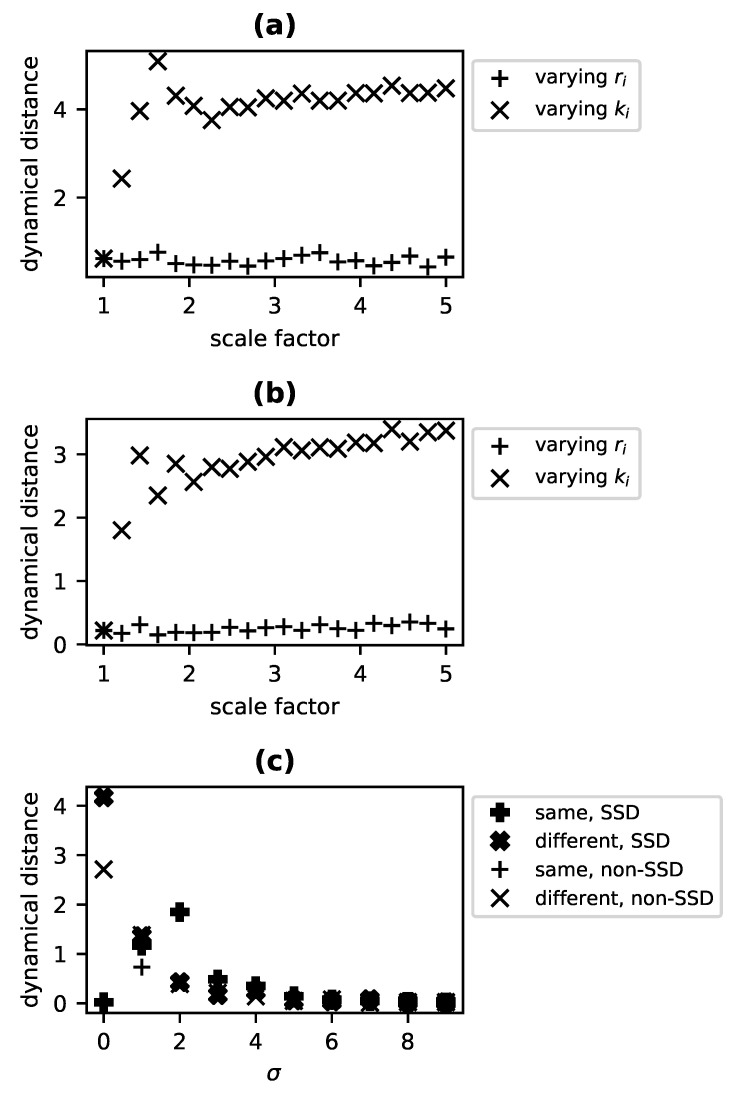
(**a**) Dynamical distance $D_{D}$ between a stochastic, two-species Lotka–Volterra system with $\vec{r}$, $\vec{k}$, and α as in Figure 2 and one for which $\vec{r}$ is multiplied by the indicated scale factor (+), keeping both systems in the same dynamical kind, or for which $\vec{k}$ is varied (×), moving the systems into increasingly distinct dynamical kinds. (**b**) The same comparison as in (**a**) except that only the average of number of species in each system (a partial set of variables that is not SSD) is provided for computing $D_{D}$. (**c**) Dynamical distance between stochastic Lotka–Volterra systems of the same dynamical kind ($\vec{r}$, $\vec{k}$, and α as in (**a**) for one system, and $\vec{r}$ doubled for the other) described with an SSD (+) or non-SSD (+) set of variables, and between systems in different dynamical kinds ($\vec{r}$, $\vec{k}$, and α as in (**a**) for one system, and $\vec{k}$ doubled for the other) as a function of the parameter σ which scales the Brownian term in the governing stochastic differential equation (higher σ corresponds to greater stochasticity, approaching a pure Brownian process as σ→∞).

We then numerically integrated the two-species version of this system in order to reproduce one of the basic tests described in Section 4.1, namely the test of sensitivity to the sameness of dynamical kind, in the context of stochastic systems in SSD and partially observed circumstances. As in the experiments of Section 4.1, a reference system with $\vec{r}=\left[1,2\right],\vec{k}=\left[100,100\right]$ was compared with either a system for which the growth rates had been multiplied by a scale factor (which does not impact dynamical kind for this stochastic system), or a system for which the carrying capacities were multiplied by the scale factor (which results in increasingly distinct dynamical kinds). For these stochastic experiments, ten replicates were included in each of the untransformed and transformed sets of time series (rather than the single replicates used for state-determined systems). The dynamical distance $D_{D}$ as a function of the value of the scale factor for the two conditions (same dynamical kinds vs. different), depicted in Figure 4a, exhibits the expected pattern, identical with that observed for the state-determined Lotka–Volterra system: varying $\vec{r}$ results in a constant $D_{D}\approx 0$ while varying $\vec{k}$ shows a rapid, apparently asymptotic increase in $D_{D}$. This test was repeated except only the average over species populations was provided at each time step, amounting to a one-dimensional, non-SSD set of variables. Nonetheless, when processed with the procedure indicated in Section 3.4.3, the same trend was apparent (Figure 4b).

Finally, we examined the behavior of $D_{D}$ as the degree of stochasticity is increased. Specifically, for 10 values of σ between 0 and 9, we generated 50 untransformed and transformed time series for three two-species systems: a reference system A for which $\vec{r}=\left[1,2\right],\vec{k}=\left[100,100\right]$, a system B of the same dynamical kind for which $\vec{r}=\left[2,4\right],$$\vec{k}=\left[100,100\right]$, and a system C of a distinct dynamical kind for which $\vec{r}=\left[1,2\right],$$\vec{k}=\left[200,200\right]$. We then computed $D_{D}$ for system A and system B (SSD systems of the same dynamical kind; ✚ marks in Figure 4c) and for Systems A and C (of different dynamical kind; ✖ marks in Figure 4c). When σ=0, the system is state-determined and we merely replicate the findings of Section 4.1: for systems of the same kind, $D_{D}\approx 0$ and systems of differing kinds, $D_{D}\approx 4$. As σ increases, however, these differences diminish. One would expect that as σ→∞ the systems would all behave as Brownian process and thus converge on a distance of $D_{D}=0$. This is clearly the case, with all values of $D_{D}$ near 0 for σ≥6.

A similar pattern is obtained when non-SSD systems are considered. Specifically, we ran the same experiment for the same values of σ but provided only the mean value of species populations for computing $D_{D}$. Though the distances are somewhat suppressed for low values of σ, the metric $D_{D}$ clearly distinguishes between systems of different dynamical kinds (+ marks in Figure 4c) while systems of the same dynamical kind (× marks in Figure 4c) exhibit distances near 0, and $D_{D}$ approaches 0 for all pairwise comparisons as increasing σ results in a pure Brownian process.

### 4.7. Change Detection

**Figure 5:**
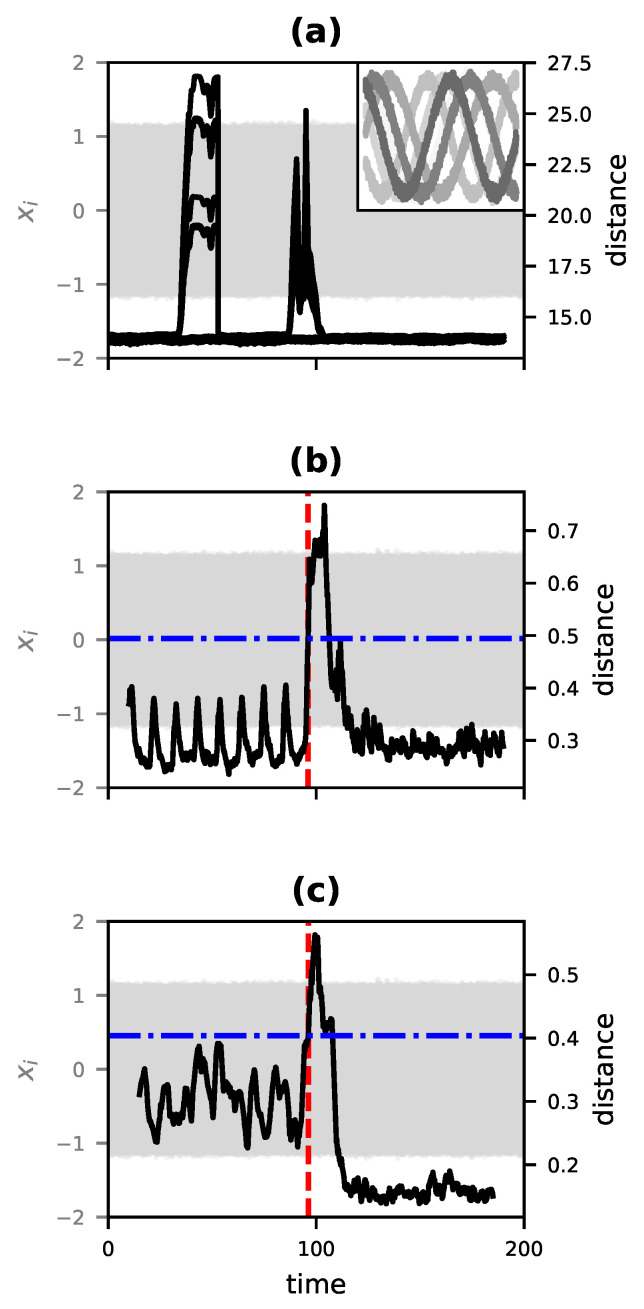
Comparison of change detection methods for a six-variable (3 phase-oscillator) Kuramoto system that transitions from uncoupled to weakly coupled over a short interval centered at 100 time units. (**a**) The matrix profile for each variable as a function of time (black lines); time series for all six variables are shown in the background in light gray, and over a short span of time in the inset. (**b**) For the same system and data as (**a**), the dynamical distance between two moving windows symmetrically arranged around each time is shown in black. The three standard deviation threshold for change detection is depicted as a dashed blue line while the vertical red dashed line indicates a detected change in dynamics. (**c**) The moving-window dynamical distance is shown (black line) for data from the same Kuramoto system but for which only one rectangular coordinate is provided from each phase oscillator (shown in light gray). The detection threshold is depicted as a dashed horizontal blue line, and the detected change event shown with a vertical dashed red line.

One of the principal applications of a general metric of dynamical similarity is the detection of shifts in the causal structure governing the behavior of a system. By computing the dynamical distance between time-lagged fragments of a time series as described in detail in Section 3.5, we can expect to find peaks of dissimilarity when the fragments differ in their underlying generative dynamics. This indicates that a structural change has occurred between the time of the first and second fragment. To test the specific approach described in Section 3.5, we simulated a three-member Kuramoto phase-oscillator system [82], generalized as:

(13) $$\frac{d\theta_{i}}{dt}=\omega_{i}+\frac{K}{N}\sum_{j=0}^{N}sin\left(\theta_{j}-\theta_{i}\right),i=0\ldots N\;$$

where $\theta_{i}$ is the phase of the *i*th oscillator, $\omega_{i}$ is that oscillator’s natural frequency, *N* is number of oscillators in the system, and *K*, the coupling coefficient, determines how much the difference in angle between oscillators affects an oscillator’s future state. For this experiment, the three-member Kuramoto system was numerically integrated for 200 time units (with a step size of $5\times 10^{-4}$), and began with a coupling value of K=1. From 95 time units to 105, the value of *K* is decreased linearly until at t=105 the oscillators are completely uncoupled (K=0) and so cannot influence one another. The data are then converted into rectangular coordinates, recording the sine and cosine of each phase, resulting in a 6-dimensional time series. A short segment of this series is shown in the inset of Figure 5a, and the full time series for all six variables is shown in light gray in all three panels of Figure 5.

As a benchmark for comparison, we computed the multidimensional matrix profile for the time series using the Stumpy Python package [83]. The result is shown as the six black curves in Figure 5a, and clearly demonstrates a false positive at a point (centered around t≈40) well before the actual start of the transition in causal structure at t=95. For using the rolling-window method of Section 3.5, we used a window size of $2\times 10^{4}$ time steps (10 time units) with the leading window and trailing window separated by 100 time steps (0.05 time units). At each step for which $D_{D}$ was computed, the time series of each window was divided into 200 fragments and then processed as described in Section 3.4.2. The resulting time series of $D_{D}$ values was smoothed as a rolling average with a window width of 50 time steps (0.025 time units). The result is shown as the black curve in Figure 5b. The initial $6\times 10^{4}$ time steps (30 units of simulated time) was assumed to derive from a constant dynamical structure and was used as a reference series to set a threshold for anomalously high values of $D_{D}$ that would indicate a change in dynamics. The dashed blue horizontal line shows this threshold, set at 3 standard deviations of the values of $D_{D}$ for the reference series. The red dashed vertical lines indicate the location at which $D_{D}$ crosses this anomaly threshold. This corresponds very closely with the onset of the transition at t=95.

To assess the efficacy of using $D_{D}$ for change detection in the more realistic scenario of a partially observed system, we repeated this experiment but discarded three of the six rectangular coordinates describing the system (preserving only the cosines of the phases). In order to compensate for the lost information, we increased the window width to $3\times 10^{4}$ time steps (15 time units). As shown in Figure 5c, the only change detected again occurs at the actual transition point at t=95.

## 5. Discussion

The concept of a degree of dynamical similarity implicitly underwrites methods for solving a variety of problems, including detection of structural change, model selection and validation, and the discrimination of nonlinearity, effective order, and chaos in a system of interest. While there exist a range of bespoke metrics, measures, and methods for probing or comparing each of these aspects for one or more systems of interest, we propose the first generic metric of dynamical similarity sensitive to all of these aspects.

The dynamical distance, $D_{D}$ is constructed of two parts. In the first place, we define a special cumulative probability density, $cdf^{*}$, for each dynamical system we wish to compare, such that the density in question differs if and only if the two dynamical systems differ with respect to their underlying dynamical symmetries. The latter are physical transformations of a system that commute with its time evolution, and are diagnostic of the underlying causal structure that generates the dynamics. Thus, the density $cdf^{*}$ is an indicator of causal structure. The second component of $D_{D}$ is the choice of a suitable metric for comparing $cdf^{*}$ between two systems. For this, we deploy the energy distance defined by [75].

We have demonstrated that $D_{D}$ is an indicator of the degree of similarity of the so-called dynamical kinds to which two systems belong. Specifically, $D_{D}=0$ if and only two systems belong to the same dynamical kind and thus share a portion of their causal structure. More importantly, the use of $D_{D}$ to assess dynamical kind improves upon the binary test of [73] by providing a degree to which two systems differ in dynamical kind and thus underlying causal structure. We have also demonstrated that $D_{D}$ is sensitive to each of the traditional dynamical features mentioned above: nonlinearity, chaos, and effective order. Specifically, $D_{D}$ increases as two systems diverge in character along one of these dimensions, e.g., with respect to the degree of nonlinearity or the presence of chaos.

The proposed metric $D_{D}$ offers a number of unique advantages when it comes to detecting differences in these dynamical features from time series data. The metric is model free and domain general. It works in the presence of significant sampling noise, and the very same metric applies to both state-determined and stochastic systems. A disadvantage, however, lies in its comparative nature and the fact that a mere difference in $D_{D}$ cannot be attributed to one or another aspect of dynamics (or the causal structure generating the dynamics) without additional information. However, with suitable reference systems—whether physical or modeled—or with assumptions about the plausible class of models, this can be overcome. For instance, divergence in $D_{D}$ from a linear reference model for a system known to be nonchaotic and first order suggests nonlinearity. This approach requires no stronger assumptions than the specialist methods for detecting, e.g., linearity, and comes with the advantage of robustness to noise and of having a single tool equally applicable to state-determined and stochastic dynamics.

**Figure 6:**
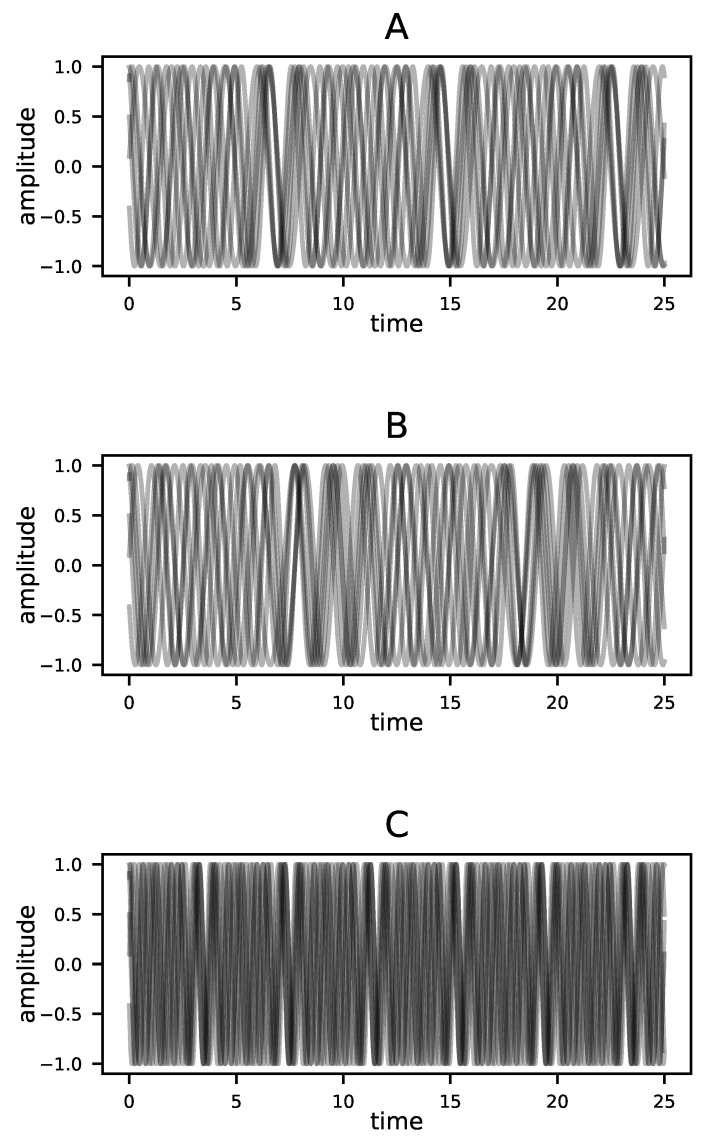
Time series from three different Kuramoto phase oscillator systems (see Equation (13)) for which each oscillator phase $\theta_{i}$ is represented by a pair of amplitudes $\sin(\theta_{i})$ and $\cos(\theta_{i})$. Despite the superficial resemblance between the time series for systems A and B, the oscillators in System B are uncoupled. Systems A and C are structurally identical (every oscillator influences every other), and differ only in the set of natural frequencies, ω.

The greatest advantage of $D_{D}$ is that it reflects structural features of the dynamics, whether deterministic or stochastic, and is otherwise indifferent to the shape or statistical properties of time series. To make this point clear, consider time series from three different Kuramoto oscillator systems, as shown in Figure 6. Each system, A, B, and C, comprises three phase oscillators governed by the equation,

(14) $$\frac{d\theta_{i}}{dt}=\omega_{i}+\frac{K}{3}\sum_{j=1}^{3}\sin\left(\theta_{j}-\theta_{i}\right),$$

where *i* ranges from 1 to 3 [84]. The figure shows each phase, $\theta_{i}$, in rectangular coordinates as a pair of curves corresponding to $\sin(\theta_{i})$ and $\cos(\theta_{i})$ as functions of time.

For Systems A and C, K=1 and the oscillators are coupled. For System C, the natural frequencies $\omega_{i}$ are twice the corresponding values for System A. However, the causal structure is the same; each oscillator influences every other oscillator. For System B, on the other hand, K=0 and the three oscillators are independent; none influences any other. Yet in terms of the statistics and gross appearance of trajectories, System C stands out as the exception. The dynamical distance proposed here is sensitive to the causal structure and not the appearance of the trajectories. Table 1 shows the values of $D_{D}$ for each pairwise comparison of the three systems, computed using the single time series given for each system and the methods described in Section 3.4.2. The distances between A and B and between B and C are an order of magnitude greater than that between A and C. In other words, the dynamical distance $D_{D}$ sees A and C, the systems with coupled oscillators, as similar and System B, which has the aberrant causal structure, as the standout. The second column of the table shows that this is not a matter of simply comparing time series statistics; the difference in mean values of the systems would suggest that A and B are most similar. More compellingly, the third column shows the minimum value of the globalized distance (the summed l2-norm normalized by path length) between each pair of time series after dynamical time warping using the dtw-python package [85]. This method, which is effective at detecting the relative similarity of time series shapes, identifies Systems A and B as the most similar. This is not wrong. Rather it is the correct answer to a question different from that which $D_{D}$ addresses. The dynamical distance is concerned with similarity in the underlying dynamics, not with the similarity of particular time series those dynamics happen to produce.

There remain a number of open questions not addressed by the results reported here. It is unclear how best to leverage simultaneously the strengths of a general metric like $D_{D}$ and the power of specialized tests. It should be possible to systematically bootstrap model identification by using $D_{D}$ to rapidly and robustly identify systems or models with similar dynamics and then deploying more powerful but limited specialized methods for determining, e.g., the effective order needed. Additionally, the methods described in Section 3.4.4 for working with passively obtained data (in circumstances where intervening on the system or controlling boundary conditions is impossible) rely on metaparameters that must be tuned. How to do this efficiently, both in terms of computational complexity and the amount of data, remains to be determined. There are also a variety of questions concerning stochastic dynamical systems. It is known that the type of noise (additive or multiplicative) in a stochastic differential equation can dramatically shape gross features such as the onset of chaos and the types of bifurcations as well as the detailed evolution of probability densities over time. The dynamical symmetries that underwrite the distance $D_{D}$ are shaped by these time evolutions. However, the relative sensitivity of $D_{D}$ to the two types of noise is unknown, as is the ability to distinguish such dynamical noise, i.e., stochastic elements in the generating process of a time series, from noise in the measurement of a system. A likely candidate for investigating these questions in simulation involves numerically extracting the time evolution of probability density functions for a given system of stochastic differential equations using the Fokker–Planck equation [6]. However, it remains uncertain whether these methods would suggest a means of using $D_{D}$ to distinguish the underlying dynamical differences between systems with different types of dynamical noise from empirically given time series.

Despite the aforementioned unknowns, the proposed metric is immediately useful for a range of applications. As demonstrated in Figure 3, the dynamical distance $D_{D}$ can be used to systematically identify regions of the parameter space of a family of models with a common dynamical character, sometimes with surprising discontinuities. For instance, there are a scattering of points in the region 0.2<α<0.4,0.2<β<0.6 that are similar in dynamical behavior to versions of the four-species Lotka–Volterra model with radically different parameterizations (α≈1). Similarly, $D_{D}$ can be used with empirical time series data to cluster physical systems with unknown underlying causal structure into classes likely to be described by similar dynamical models. This facilitates development of such models. Additionally, $D_{D}$ can be used to validate models—especially stochastic models—when the ground truth is unknown and only time series from a target system are available, similar to the related approach of [69]. Insofar as time series produced with the model diverge in $D_{D}$ from time series measured from the target system, one can determine whether a model correctly captures the causal structure regardless of how well the model is able to mimic any particular trajectory. Finally, perhaps the most promising application of the dynamical distance metric is detecting structural change. We demonstrated using fully and partially observed Kuramoto phase oscillator systems (Section 4.7) that $D_{D}$ can identify points in a time series at which the underlying generative dynamics changes. The dynamical distance is uniquely apt for detecting a genuine change in the underlying causal structure rather than a shift to a previously unobserved portion of the system’s phase space. This is precisely what is needed to identify, e.g., a change in ecosystem dynamics portending collapse or recovery, or a shift in the behavior of a volcano system suggesting worrisome internal structural changes. This list is only suggestive; there are myriad uses for a general metric of dynamical similarity that is tied to underlying causal structure, can be inferred from passively acquired time series observations, is robust under measurement noise, and is applicable to state-determined and stochastic dynamics alike. 

## Figures and Tables

**Table 1:** Distances between the systems represented in Figure 6. $D_{D}$ is the dynamical distance computed between the indicated systems in each row using the methods described in Section 3.4.2 for singly sampled observational time series. Δμ is the difference in mean values between the indicated time series, while DTW is the minimum global distance (normalized by path length) obtained after dynamic time warping to align the indicated time series. Minimum values in each column are bold-faced.

Systems Compared	$D_{D}$	Δμ	DTW
A, B	3.38	**0.014**	**294**
A, C	**0.575**	0.032	425
B, C	3.36	0.028	426

## Data Availability

A Python package implementing all of the methods reported here is available under an MIT license from https://github.com/jantzen/eugene (accessed on 6 September 2021). Scripts for conducting all tests reported in the text and generating the figures presented can be obtained from https://github.com/jantzen/distance_metric (accessed on 6 September 2021). Because the numerical experiments reported involve a stochastic element, repeating the experiments will not give exactly the results reported. To recreate the exact analyses reported here, the specific data we used can be obtained from https://doi.org/10.7294/16587179 (accessed on 6 September 2021).
